# Methyl 3,4-bis­(cyclo­propyl­meth­oxy)benzoate

**DOI:** 10.1107/S1600536811013158

**Published:** 2011-04-13

**Authors:** Xian-Chao Cheng, Jing-Jing Hou, Cheng-Zhi Xie, Run-Ling Wang, Wei-Ren Xu

**Affiliations:** a School of Pharmaceutical Sciences, Tianjin Medical University, Tianjin 300070, People’s Republic of China; b Tianjin Key Laboratory of Molecular Design and Drug Discovery, Tianjin Institute of Pharmaceutical Research, Tianjin 300193, People’s Republic of China

## Abstract

The title compound, C_16_H_20_O_4_, was obtained unintentionally as the byproduct of an attempted synthesis of methyl 3-(cyclo­propyl­meth­oxy)-4-hy­droxy­benzoate. In the crystal, the mol­ecules are linked by inter­molecular C—H⋯O inter­actions.

## Related literature

For the preparation, see: Bose *et al.* (2005 ▶). For a similar structure, see: Hou *et al.* (2010 ▶).
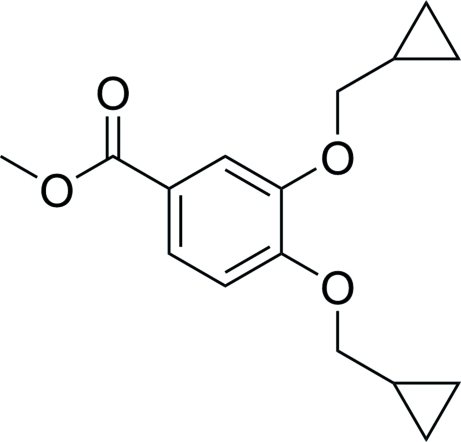


## Experimental

### 

#### Crystal data

C_16_H_20_O_4_
*M* *_r_* = 276.33
Orthorhombic, 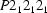

*a* = 4.9018 (8) Å
*b* = 15.543 (2) Å
*c* = 18.846 (2) Å
*V* = 1435.9 (3) Å^3^
*Z* = 4
Mo *K*α radiation
μ = 0.09 mm^−1^
*T* = 113 K
0.22 × 0.20 × 0.18 mm

#### Data collection

Rigaku Saturn724 CCD diffractometer
Absorption correction: multi-scan (*REQAB*; Jacobson, 1998 ▶) *T*_min_ = 0.891, *T*_max_ = 0.984
20068 measured reflections
3852 independent reflections
3300 reflections with *F* ^2^ > 2.0σ(*F* ^2^)
*R*_int_ = 0.034

#### Refinement

*R*[*F* ^2^ > 2σ(*F* ^2^)] = 0.041
*wR*(*F* ^2^) = 0.114
*S* = 1.06
3852 reflections
182 parameters
H-atom parameters constrained
Δρ_max_ = 0.57 e Å^−3^
Δρ_min_ = −0.19 e Å^−3^

### 

Data collection: *CrystalClear-SM Expert* (Rigaku, 2009 ▶); cell refinement: *CrystalClear-SM Expert*; data reduction: *CrystalClear-SM Expert*; program(s) used to solve structure: *SHELXS97* (Sheldrick, 2008 ▶); program(s) used to refine structure: *SHELXL97* (Sheldrick, 2008 ▶); molecular graphics: *CrystalStructure* (Rigaku, 2009 ▶); software used to prepare material for publication: *CrystalStructure*.

## Supplementary Material

Crystal structure: contains datablocks global, I. DOI: 10.1107/S1600536811013158/jh2279sup1.cif

Structure factors: contains datablocks I. DOI: 10.1107/S1600536811013158/jh2279Isup2.hkl

Additional supplementary materials:  crystallographic information; 3D view; checkCIF report

## Figures and Tables

**Table 1:** Hydrogen-bond geometry (Å, °)

*D*—H⋯*A*	*D*—H	H⋯*A*	*D*⋯*A*	*D*—H⋯*A*
C12—H12B⋯O3^i^	0.99	2.55	3.4073 (18)	145

Symmetry code: (i) 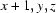.

**Fig. 1.**
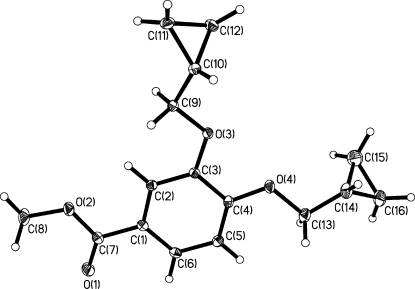
The molecular structure of the title compound, with atom labels and 30% probability displacement ellipsoids, and H atoms are shown as small spheres of arbitrary radius.

**Fig. 2.**
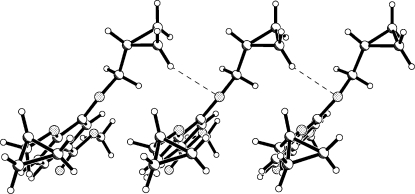
The packing of the title compound, showing the one-dimensional structure,with intermolecular hydrogen bonds (dashed lines); for clarity H atoms have been omitted.

## supplementary crystallographic information

### Comment

Roflumilast is an effective phosphodiesterase-4 inhibitor (PDE4 inhibitor), which can be used in the treatment of asthma, inflammation, bronchitis, allergy and other disorders related to immune system, heart and kidney. During the development of our own PDE4 inhibitors, roflumilast was synthesized as the positive control in the bioactivity screening, and the title compound,methyl 3,4-bis(cyclopropylmethoxy)benzoate, was a byproduct during preparation of the intermediate methyl 3-(cyclopropylmethoxy)-4-hydroxybenzoate.The crystallographic analysis of the title compound is done to confirm the chemical structure of the title compound. In the title compound, all bond lengths and angles are normal and in a good agreement with those reported previously (Hou, *et al.*, 2010). In the crystal structure, the hydroxy groups are involved in the formation of intermolecular C—H···O hydrogen bonds(Tab 1), which link the molecules related by translation along axis b into one-dimensional chains.

### Experimental

A mixture of 3,4-dihydroxy methyl benzoate (1.68 g, 10 mmol) and potassium carbonate (2.76 g, 20 mmol) in acetone (50 ml) was added with a solution of cyclopropyl methyl bromide (1.35 g, 10 mmol) in acetone (50 ml). The reaction mixture was stirred at 40 °C for 18 h, and then was filtered. The filtrate was evaporated on a rotary evaporator to get the dried solid, which was then purified by flash column chromatography to obtain methyl 3-(cyclopropylmethoxy)-4-hydroxybenzoate, methyl 4-(cyclopropylmethoxy)-3-hydroxybenzoate, and the title compound methyl 3,4-bis(cyclopropylmethoxy)benzoate(Bose,*et al.*,2005).

Crystals suitable for X-ray diffraction were obtained through slow evaporation of a solution of the pure title compound in ethyl acetate/n-hexane (1/10 by volume).

### Refinement

H atoms were positioned geometrically (C—H = 0.95–1.00 Å) and refined as riding, with *U*_iso_(H) = 1.2 *U*_eq_ of the parent atom.

### Crystal data

**Table:**

C_16_H_20_O_4_	*F*(000) = 592.00
*M**_r_* = 276.33	*D*_x_ = 1.278 Mg m^−^^3^
Orthorhombic, *P*2_1_2_1_2_1_	Mo *K*α radiation, λ = 0.71075 Å
Hall symbol: P 2ac 2ab	Cell parameters from 6064 reflections
*a* = 4.9018 (8) Å	θ = 1.3–31.4°
*b* = 15.543 (2) Å	µ = 0.09 mm^−^^1^
*c* = 18.846 (2) Å	*T* = 113 K
*V* = 1435.9 (3) Å^3^	Prism, colorless
*Z* = 4	0.22 × 0.20 × 0.18 mm

### Data collection

**Table:**

Rigaku Saturn724 CCD diffractometer	3300 reflections with *F*^2^ > 2.0σ(*F*^2^)
Detector resolution: 14.222 pixels mm^-1^	*R*_int_ = 0.034
ω scans	θ_max_ = 29.1°
Absorption correction: multi-scan (REQAB; Jacobson, 1998)	*h* = −6→6
*T*_min_ = 0.891, *T*_max_ = 0.984	*k* = −21→21
20068 measured reflections	*l* = −25→25
3852 independent reflections	

### Refinement

**Table:**

Refinement on *F*^2^	Secondary atom site location: difference Fourier map
*R*[*F*^2^ > 2σ(*F*^2^)] = 0.041	Hydrogen site location: inferred from neighbouring sites
*wR*(*F*^2^) = 0.114	H-atom parameters constrained
*S* = 1.06	*w* = 1/[σ^2^(*F*_o_^2^) + (0.0778*P*)^2^] where *P* = (*F*_o_^2^ + 2*F*_c_^2^)/3
3852 reflections	(Δ/σ)_max_ = 0.001
182 parameters	Δρ_max_ = 0.57 e Å^−^^3^
0 restraints	Δρ_min_ = −0.19 e Å^−^^3^
Primary atom site location: structure-invariant direct methods	

### Special details

**Table:**

Geometry. ENTER SPECIAL DETAILS OF THE MOLECULAR GEOMETRY
Refinement. Refinement was performed using all reflections. The weighted *R*-factor (*wR*) and goodness of fit (*S*) are based on *F*^2^. *R*-factor (gt) are based on *F*. The threshold expression of *F*^2^ > 2.0 σ(*F*^2^) is used only for calculating *R*-factor (gt).

### Fractional atomic coordinates and isotropic or equivalent isotropic displacement parameters (Å^2^)

**Table:**

	*x*	*y*	*z*	*U*_iso_*/*U*_eq_	
O(1)	0.3872 (2)	0.69719 (7)	0.66936 (5)	0.0321	
O(2)	0.6968 (2)	0.59026 (7)	0.66908 (5)	0.0306	
O(3)	0.7825 (2)	0.48335 (5)	0.42141 (5)	0.0209	
O(4)	0.4320 (2)	0.56811 (6)	0.34625 (4)	0.0250	
C(1)	0.4862 (3)	0.62225 (8)	0.56094 (7)	0.0192	
C(2)	0.6558 (3)	0.56094 (8)	0.52884 (7)	0.0184	
C(3)	0.6319 (3)	0.54351 (8)	0.45719 (6)	0.0174	
C(4)	0.4378 (3)	0.58918 (8)	0.41612 (7)	0.0193	
C(5)	0.2695 (3)	0.64967 (8)	0.44863 (7)	0.0213	
C(6)	0.2930 (3)	0.66574 (8)	0.52102 (7)	0.0211	
C(7)	0.5139 (3)	0.64194 (8)	0.63788 (7)	0.0216	
C(8)	0.7487 (4)	0.60502 (11)	0.74361 (7)	0.0406	
C(9)	0.9395 (3)	0.42507 (8)	0.46452 (6)	0.0199	
C(10)	1.0118 (3)	0.34763 (8)	0.42075 (7)	0.0205	
C(11)	1.2662 (3)	0.29938 (9)	0.44212 (8)	0.0262	
C(12)	1.2614 (3)	0.35005 (9)	0.37423 (7)	0.0246	
C(13)	0.2283 (3)	0.61039 (9)	0.30217 (7)	0.0302	
C(14)	0.2718 (4)	0.57919 (10)	0.22787 (7)	0.0330	
C(15)	0.5052 (4)	0.61632 (12)	0.18752 (10)	0.0442	
C(16)	0.2225 (4)	0.64137 (13)	0.16830 (8)	0.0428	
H(2)	0.7878	0.5312	0.5565	0.022*	
H(5)	0.1381	0.6800	0.4213	0.026*	
H(6)	0.1765	0.7066	0.5431	0.025*	
H(8A)	0.5750	0.6078	0.7693	0.049*	
H(8B)	0.8469	0.6595	0.7495	0.049*	
H(8C)	0.8593	0.5578	0.7626	0.049*	
H(9A)	0.8319	0.4073	0.5065	0.024*	
H(9B)	1.1078	0.4538	0.4813	0.024*	
H(10)	0.8558	0.3120	0.4033	0.025*	
H(11A)	1.3747	0.3225	0.4821	0.031*	
H(11B)	1.2633	0.2358	0.4386	0.031*	
H(12A)	1.2558	0.3176	0.3291	0.030*	
H(12B)	1.3671	0.4043	0.3725	0.030*	
H(13A)	0.2504	0.6736	0.3045	0.036*	
H(13B)	0.0423	0.5955	0.3186	0.036*	
H(14)	0.2271	0.5175	0.2183	0.040*	
H(15A)	0.6201	0.6597	0.2118	0.053*	
H(15B)	0.6047	0.5779	0.1546	0.053*	
H(16A)	0.1462	0.6184	0.1235	0.051*	
H(16B)	0.1615	0.7002	0.1807	0.051*	

### Atomic displacement parameters (Å^2^)

**Table:**

	*U*^11^	*U*^22^	*U*^33^	*U*^12^	*U*^13^	*U*^23^
O(1)	0.0398	0.0311	0.0253	0.0080	0.0001	−0.0084
O(2)	0.0391	0.0348	0.0179	0.0103	−0.0046	−0.0070
O(3)	0.0251	0.0207	0.0170	0.0073	−0.0011	0.0001
O(4)	0.0346	0.0232	0.0170	0.0084	−0.0041	0.0008
C(1)	0.0205	0.0176	0.0194	−0.0020	0.0025	−0.0008
C(2)	0.0171	0.0187	0.0194	−0.0004	−0.0002	−0.0003
C(3)	0.0192	0.0152	0.0178	0.0006	0.0017	−0.0000
C(4)	0.0228	0.0182	0.0170	−0.0013	−0.0017	0.0020
C(5)	0.0222	0.0188	0.0229	0.0010	−0.0014	0.0024
C(6)	0.0213	0.0185	0.0235	0.0012	0.0042	−0.0012
C(7)	0.0243	0.0197	0.0208	−0.0031	0.0009	−0.0025
C(8)	0.0575	0.0441	0.0202	0.0155	−0.0122	−0.0113
C(9)	0.0213	0.0205	0.0179	0.0038	−0.0004	0.0030
C(10)	0.0189	0.0203	0.0222	0.0011	0.0009	0.0013
C(11)	0.0251	0.0244	0.0291	0.0071	0.0026	0.0046
C(12)	0.0247	0.0255	0.0236	0.0017	0.0060	0.0002
C(13)	0.0337	0.0335	0.0235	0.0069	−0.0052	0.0038
C(14)	0.0389	0.0350	0.0251	0.0013	−0.0049	0.0053
C(15)	0.0457	0.0498	0.0371	0.0028	0.0038	0.0098
C(16)	0.0510	0.0542	0.0230	0.0148	−0.0043	0.0114

### Geometric parameters (Å, °)

**Table:**

O(1)—C(7)	1.2143 (17)	C(15)—C(16)	1.484 (3)
O(2)—C(7)	1.3397 (11)	C(2)—H(2)	0.950
O(2)—C(8)	1.4458 (19)	C(5)—H(5)	0.950
O(3)—C(3)	1.3692 (14)	C(6)—H(6)	0.950
O(3)—C(9)	1.4397 (10)	C(8)—H(8A)	0.980
O(4)—C(4)	1.3572 (17)	C(8)—H(8B)	0.980
O(4)—C(13)	1.4558 (13)	C(8)—H(8C)	0.980
C(1)—C(2)	1.4017 (18)	C(9)—H(9A)	0.990
C(1)—C(6)	1.3856 (15)	C(9)—H(9B)	0.990
C(1)—C(7)	1.488 (3)	C(10)—H(10)	1.000
C(2)—C(3)	1.382 (3)	C(11)—H(11A)	0.990
C(3)—C(4)	1.4169 (14)	C(11)—H(11B)	0.990
C(4)—C(5)	1.3930 (18)	C(12)—H(12A)	0.990
C(5)—C(6)	1.392 (3)	C(12)—H(12B)	0.990
C(9)—C(10)	1.502 (2)	C(13)—H(13A)	0.990
C(10)—C(11)	1.5098 (14)	C(13)—H(13B)	0.990
C(10)—C(12)	1.506 (2)	C(14)—H(14)	1.000
C(11)—C(12)	1.5026 (17)	C(15)—H(15A)	0.990
C(13)—C(14)	1.497 (3)	C(15)—H(15B)	0.990
C(14)—C(15)	1.490 (3)	C(16)—H(16A)	0.990
C(14)—C(16)	1.5009 (15)	C(16)—H(16B)	0.990
			
O(1)···C(6)	2.875 (2)	H(6)···C(1)^viii^	3.4340
O(1)···C(8)	2.6738 (14)	H(6)···C(2)^iv^	3.4221
O(2)···C(2)	2.6895 (19)	H(6)···C(4)^viii^	3.4694
O(3)···O(4)	2.5873 (12)	H(6)···C(5)^viii^	2.9995
O(3)···C(12)	3.2546 (14)	H(6)···C(6)^viii^	2.9886
O(4)···C(15)	3.1046 (16)	H(6)···H(2)^iv^	3.3350
C(1)···C(4)	2.787 (3)	H(6)···H(5)^viii^	3.2433
C(2)···C(5)	2.7880 (15)	H(6)···H(5)^i^	2.9456
C(2)···C(9)	2.804 (2)	H(6)···H(6)^viii^	3.2354
C(3)···C(6)	2.7955 (18)	H(6)···H(6)^i^	3.2354
C(5)···C(13)	2.834 (3)	H(8A)···O(3)^ii^	3.2735
O(1)···C(13)^i^	3.468 (4)	H(8A)···C(10)^ii^	2.9685
O(2)···C(15)^ii^	3.545 (2)	H(8A)···C(12)^ii^	2.6574
O(3)···C(5)^iii^	3.556 (2)	H(8A)···C(14)^ix^	3.4558
O(3)···C(12)^iv^	3.407 (2)	H(8A)···H(8C)^iv^	3.5954
O(4)···C(12)^iv^	3.5306 (19)	H(8A)···H(10)^ii^	2.8381
C(2)···C(6)^iii^	3.526 (3)	H(8A)···H(12A)^ii^	2.2901
C(3)···C(5)^iii^	3.538 (3)	H(8A)···H(12B)^ii^	2.9188
C(5)···O(3)^iv^	3.556 (3)	H(8A)···H(12B)^v^	3.3617
C(5)···C(3)^iv^	3.538 (3)	H(8A)···H(13B)^ix^	3.3436
C(6)···C(2)^iv^	3.526 (3)	H(8A)···H(14)^ix^	2.6278
C(8)···C(12)^ii^	3.578 (3)	H(8A)···H(16B)^i^	3.1585
C(8)···C(12)^v^	3.509 (7)	H(8B)···O(1)^iii^	3.1047
C(12)···O(3)^iii^	3.407 (4)	H(8B)···C(12)^v^	3.0389
C(12)···O(4)^iii^	3.531 (3)	H(8B)···C(16)^i^	3.5151
C(12)···C(8)^vi^	3.578 (5)	H(8B)···H(10)^ii^	3.0967
C(12)···C(8)^vii^	3.509 (5)	H(8B)···H(12A)^ii^	3.3323
C(13)···O(1)^viii^	3.468 (3)	H(8B)···H(12A)^v^	2.4836
C(15)···O(2)^vi^	3.545 (3)	H(8B)···H(12B)^v^	2.8849
C(15)···C(16)^iii^	3.556 (4)	H(8B)···H(13A)^i^	2.8266
C(16)···C(15)^iv^	3.556 (3)	H(8B)···H(14)^ii^	3.5027
O(1)···H(6)	2.5982	H(8B)···H(15A)^i^	3.1979
O(1)···H(8A)	2.5145	H(8B)···H(16B)^i^	2.7043
O(1)···H(8B)	2.7752	H(8C)···O(3)^ii^	3.1385
O(2)···H(2)	2.3550	H(8C)···O(4)^ii^	2.7133
O(3)···H(2)	2.6517	H(8C)···C(12)^v^	3.1519
O(3)···H(10)	2.7092	H(8C)···C(13)^ii^	3.3878
O(3)···H(12B)	3.2512	H(8C)···C(14)^ii^	2.8694
O(4)···H(5)	2.6652	H(8C)···C(15)^ii^	3.1254
O(4)···H(14)	2.7276	H(8C)···H(8A)^iii^	3.5954
O(4)···H(15A)	3.0496	H(8C)···H(10)^ii^	3.4987
C(1)···H(5)	3.2624	H(8C)···H(12A)^v^	2.9798
C(2)···H(6)	3.2736	H(8C)···H(12B)^v^	2.5373
C(2)···H(9A)	2.5735	H(8C)···H(13B)^ix^	3.2663
C(2)···H(9B)	2.9122	H(8C)···H(14)^ix^	3.2138
C(3)···H(5)	3.2890	H(8C)···H(14)^ii^	2.4852
C(3)···H(9A)	2.5109	H(8C)···H(15A)^ii^	3.5154
C(3)···H(9B)	2.7551	H(8C)···H(15B)^ii^	2.9371
C(4)···H(2)	3.2784	H(9A)···C(11)^iv^	3.4606
C(4)···H(6)	3.2707	H(9A)···C(11)^x^	3.3711
C(4)···H(13A)	2.6440	H(9A)···C(15)^ii^	3.5231
C(4)···H(13B)	2.6731	H(9A)···H(11A)^iv^	2.6402
C(5)···H(13A)	2.7429	H(9A)···H(11A)^x^	3.5855
C(5)···H(13B)	2.8201	H(9A)···H(11B)^x^	2.4769
C(6)···H(2)	3.2707	H(9A)···H(12B)^iv^	3.4008
C(7)···H(2)	2.6681	H(9A)···H(15B)^ii^	2.8173
C(7)···H(6)	2.6334	H(9A)···H(16A)^ix^	3.2426
C(7)···H(8A)	2.5499	H(9A)···H(16A)^ii^	3.4014
C(7)···H(8B)	2.6766	H(9B)···O(3)^iii^	3.5250
C(7)···H(8C)	3.1783	H(9B)···O(4)^iii^	3.4870
C(9)···H(2)	2.5055	H(9B)···C(1)^iii^	3.5419
C(9)···H(11A)	2.6830	H(9B)···C(2)^iii^	3.2847
C(9)···H(11B)	3.3775	H(9B)···C(3)^iii^	2.9578
C(9)···H(12A)	3.4221	H(9B)···C(4)^iii^	2.9243
C(9)···H(12B)	2.7392	H(9B)···C(5)^iii^	3.2049
C(11)···H(9A)	2.9697	H(9B)···C(6)^iii^	3.4973
C(11)···H(9B)	2.6293	H(9B)···H(15B)^ii^	3.4627
C(12)···H(9A)	3.3820	H(9B)···H(16A)^ii^	3.1460
C(12)···H(9B)	2.6912	H(10)···C(8)^vi^	3.3147
C(13)···H(5)	2.5315	H(10)···C(11)^iv^	2.9874
C(13)···H(15A)	2.6793	H(10)···C(11)^x^	3.4165
C(13)···H(15B)	3.3759	H(10)···C(12)^iv^	3.0231
C(13)···H(16A)	3.3933	H(10)···C(16)^xi^	3.0002
C(13)···H(16B)	2.7007	H(10)···H(8A)^vi^	2.8381
C(15)···H(13A)	2.6862	H(10)···H(8B)^vi^	3.0967
C(15)···H(13B)	3.3697	H(10)···H(8C)^vi^	3.4987
C(16)···H(13A)	2.6193	H(10)···H(11A)^iv^	2.7913
C(16)···H(13B)	3.0514	H(10)···H(11A)^x^	3.0070
H(2)···H(9A)	2.1545	H(10)···H(11B)^iv^	3.2057
H(2)···H(9B)	2.4315	H(10)···H(11B)^x^	3.1032
H(5)···H(6)	2.3396	H(10)···H(12A)^iv^	3.2582
H(5)···H(13A)	2.2709	H(10)···H(12B)^iv^	2.8516
H(5)···H(13B)	2.3861	H(10)···H(16A)^xi^	3.0514
H(9A)···H(10)	2.4474	H(10)···H(16B)^xi^	2.3527
H(9A)···H(11A)	3.0045	H(11A)···O(3)^iii^	3.3990
H(9B)···H(10)	2.9237	H(11A)···C(9)^iii^	3.2119
H(9B)···H(11A)	2.4243	H(11A)···C(10)^iii^	3.3530
H(9B)···H(11B)	3.5652	H(11A)···C(10)^xii^	3.2863
H(9B)···H(12B)	2.5324	H(11A)···C(11)^xii^	3.0519
H(10)···H(11A)	2.9497	H(11A)···C(16)^ii^	3.5854
H(10)···H(11B)	2.4153	H(11A)···H(9A)^iii^	2.6402
H(10)···H(12A)	2.4108	H(11A)···H(9A)^xii^	3.5855
H(10)···H(12B)	2.9459	H(11A)···H(10)^iii^	2.7913
H(11A)···H(12A)	2.9435	H(11A)···H(10)^xii^	3.0070
H(11A)···H(12B)	2.4248	H(11A)···H(11A)^x^	3.3981
H(11B)···H(12A)	2.4247	H(11A)···H(11A)^xii^	3.3981
H(11B)···H(12B)	2.9436	H(11A)···H(11B)^x^	3.4694
H(13A)···H(14)	2.9227	H(11A)···H(11B)^xii^	2.5855
H(13A)···H(15A)	2.5272	H(11A)···H(16A)^ii^	2.8206
H(13A)···H(16A)	3.5547	H(11B)···C(9)^xii^	3.2146
H(13A)···H(16B)	2.4089	H(11B)···C(10)^xii^	3.1923
H(13B)···H(14)	2.4211	H(11B)···C(11)^x^	3.3599
H(13B)···H(16B)	3.1209	H(11B)···C(11)^xii^	3.3805
H(14)···H(15A)	2.9345	H(11B)···C(15)^xiii^	3.2233
H(14)···H(15B)	2.3983	H(11B)···C(16)^xi^	3.4476
H(14)···H(16A)	2.4103	H(11B)···C(16)^xiii^	3.5454
H(14)···H(16B)	2.9445	H(11B)···H(9A)^xii^	2.4769
H(15A)···H(16A)	2.9283	H(11B)···H(10)^iii^	3.2057
H(15A)···H(16B)	2.4063	H(11B)···H(10)^xii^	3.1032
H(15B)···H(16A)	2.4063	H(11B)···H(11A)^x^	2.5855
H(15B)···H(16B)	2.9283	H(11B)···H(11A)^xii^	3.4694
O(1)···H(5)^i^	2.8414	H(11B)···H(11B)^x^	3.3990
O(1)···H(8B)^iv^	3.1047	H(11B)···H(11B)^xii^	3.3990
O(1)···H(12A)^ii^	3.0988	H(11B)···H(15A)^xiii^	3.1240
O(1)···H(13A)^i^	2.7280	H(11B)···H(15B)^xiii^	3.0867
O(1)···H(13B)^i^	3.3183	H(11B)···H(16A)^xi^	2.9551
O(1)···H(14)^ix^	3.5071	H(11B)···H(16B)^xi^	3.1149
O(1)···H(15A)^viii^	3.4180	H(12A)···O(1)^vi^	3.0988
O(1)···H(16B)^i^	3.5118	H(12A)···C(8)^vi^	3.1867
O(2)···H(14)^ix^	2.8256	H(12A)···C(8)^vii^	3.1528
O(2)···H(14)^ii^	3.4119	H(12A)···C(15)^xiii^	3.3551
O(2)···H(15B)^ii^	2.8026	H(12A)···H(8A)^vi^	2.2901
O(3)···H(5)^iii^	3.5191	H(12A)···H(8B)^vi^	3.3323
O(3)···H(8A)^vi^	3.2735	H(12A)···H(8B)^vii^	2.4836
O(3)···H(8C)^vi^	3.1385	H(12A)···H(8C)^vii^	2.9798
O(3)···H(9B)^iv^	3.5250	H(12A)···H(10)^iii^	3.2582
O(3)···H(11A)^iv^	3.3990	H(12A)···H(15A)^xiii^	2.6428
O(3)···H(12B)^iv^	2.5506	H(12A)···H(16B)^xi^	2.7473
O(3)···H(13B)^iii^	2.9009	H(12A)···H(16B)^xiii^	3.3943
O(4)···H(8C)^vi^	2.7133	H(12B)···O(3)^iii^	2.5506
O(4)···H(9B)^iv^	3.4870	H(12B)···O(4)^iii^	2.6136
O(4)···H(12B)^iv^	2.6136	H(12B)···C(3)^iii^	2.9854
O(4)···H(13B)^iii^	3.0664	H(12B)···C(4)^iii^	3.0090
C(1)···H(5)^i^	3.1796	H(12B)···C(8)^vii^	3.0774
C(1)···H(6)^i^	3.4340	H(12B)···C(9)^iii^	3.3140
C(1)···H(9B)^iv^	3.5419	H(12B)···C(10)^iii^	3.4040
C(2)···H(6)^iii^	3.4221	H(12B)···C(13)^iii^	3.5333
C(2)···H(9B)^iv^	3.2847	H(12B)···H(8A)^vi^	2.9188
C(2)···H(15B)^ii^	3.4133	H(12B)···H(8A)^vii^	3.3617
C(3)···H(5)^iii^	3.3346	H(12B)···H(8B)^vii^	2.8849
C(3)···H(9B)^iv^	2.9578	H(12B)···H(8C)^vii^	2.5373
C(3)···H(12B)^iv^	2.9854	H(12B)···H(9A)^iii^	3.4008
C(3)···H(13B)^iii^	3.3947	H(12B)···H(10)^iii^	2.8516
C(4)···H(6)^i^	3.4694	H(12B)···H(13B)^iii^	3.5216
C(4)···H(9B)^iv^	2.9243	H(12B)···H(14)^iii^	3.4659
C(4)···H(12B)^iv^	3.0090	H(13A)···O(1)^viii^	2.7280
C(4)···H(13B)^iii^	3.4883	H(13A)···C(7)^viii^	3.2771
C(5)···H(6)^i^	2.9995	H(13A)···C(8)^viii^	3.5579
C(5)···H(9B)^iv^	3.2049	H(13A)···H(8B)^viii^	2.8266
C(6)···H(2)^iv^	3.3092	H(13A)···H(15A)^iv^	3.5566
C(6)···H(5)^i^	3.1287	H(13B)···O(1)^viii^	3.3183
C(6)···H(6)^i^	2.9886	H(13B)···O(3)^iv^	2.9009
C(6)···H(9B)^iv^	3.4973	H(13B)···O(4)^iv^	3.0664
C(7)···H(5)^i^	3.0449	H(13B)···C(3)^iv^	3.3947
C(7)···H(13A)^i^	3.2771	H(13B)···C(4)^iv^	3.4883
C(7)···H(14)^ix^	3.1360	H(13B)···H(8A)^xiv^	3.3436
C(8)···H(10)^ii^	3.3147	H(13B)···H(8C)^xiv^	3.2663
C(8)···H(12A)^ii^	3.1867	H(13B)···H(12B)^iv^	3.5216
C(8)···H(12A)^v^	3.1528	H(13B)···H(15A)^iv^	3.0552
C(8)···H(12B)^v^	3.0774	H(14)···O(1)^xiv^	3.5071
C(8)···H(13A)^i^	3.5579	H(14)···O(2)^xiv^	2.8256
C(8)···H(14)^ix^	3.0483	H(14)···O(2)^vi^	3.4119
C(8)···H(14)^ii^	3.2335	H(14)···C(7)^xiv^	3.1360
C(8)···H(15B)^ii^	3.3790	H(14)···C(8)^xiv^	3.0483
C(8)···H(16B)^i^	3.3736	H(14)···C(8)^vi^	3.2335
C(9)···H(11A)^iv^	3.2119	H(14)···H(8A)^xiv^	2.6278
C(9)···H(11B)^x^	3.2146	H(14)···H(8B)^vi^	3.5027
C(9)···H(12B)^iv^	3.3140	H(14)···H(8C)^xiv^	3.2138
C(9)···H(15B)^ii^	3.5885	H(14)···H(8C)^vi^	2.4852
C(10)···H(8A)^vi^	2.9685	H(14)···H(12B)^iv^	3.4659
C(10)···H(11A)^iv^	3.3530	H(14)···H(15B)^iv^	3.4109
C(10)···H(11A)^x^	3.2863	H(15A)···O(1)^i^	3.4180
C(10)···H(11B)^x^	3.1923	H(15A)···C(12)^xv^	3.4231
C(10)···H(12B)^iv^	3.4040	H(15A)···C(13)^iii^	3.5184
C(10)···H(16B)^xi^	3.1033	H(15A)···C(14)^iii^	3.4443
C(11)···H(9A)^iii^	3.4606	H(15A)···C(16)^iii^	3.0775
C(11)···H(9A)^xii^	3.3711	H(15A)···H(8B)^viii^	3.1979
C(11)···H(10)^iii^	2.9874	H(15A)···H(8C)^vi^	3.5154
C(11)···H(10)^xii^	3.4165	H(15A)···H(11B)^xv^	3.1240
C(11)···H(11A)^x^	3.0519	H(15A)···H(12A)^xv^	2.6428
C(11)···H(11B)^x^	3.3805	H(15A)···H(13A)^iii^	3.5566
C(11)···H(11B)^xii^	3.3599	H(15A)···H(13B)^iii^	3.0552
C(11)···H(16B)^xi^	3.4832	H(15A)···H(16A)^iii^	3.1351
C(12)···H(8A)^vi^	2.6574	H(15A)···H(16B)^iii^	2.7897
C(12)···H(8B)^vii^	3.0389	H(15B)···O(2)^vi^	2.8026
C(12)···H(8C)^vii^	3.1519	H(15B)···C(2)^vi^	3.4133
C(12)···H(10)^iii^	3.0231	H(15B)···C(8)^vi^	3.3790
C(12)···H(15A)^xiii^	3.4231	H(15B)···C(9)^vi^	3.5885
C(12)···H(16B)^xi^	3.2856	H(15B)···C(14)^iii^	3.5496
C(13)···H(8C)^vi^	3.3878	H(15B)···C(16)^iii^	3.1951
C(13)···H(12B)^iv^	3.5333	H(15B)···H(2)^vi^	2.5642
C(13)···H(15A)^iv^	3.5184	H(15B)···H(8C)^vi^	2.9371
C(14)···H(8A)^xiv^	3.4558	H(15B)···H(9A)^vi^	2.8173
C(14)···H(8C)^vi^	2.8694	H(15B)···H(9B)^vi^	3.4627
C(14)···H(15A)^iv^	3.4443	H(15B)···H(11B)^xv^	3.0867
C(14)···H(15B)^iv^	3.5496	H(15B)···H(14)^iii^	3.4109
C(15)···H(2)^vi^	3.5201	H(15B)···H(16A)^iii^	2.7897
C(15)···H(8C)^vi^	3.1254	H(15B)···H(16B)^iii^	3.3623
C(15)···H(9A)^vi^	3.5231	H(16A)···C(15)^iv^	3.3656
C(15)···H(11B)^xv^	3.2233	H(16A)···H(2)^xiv^	3.3954
C(15)···H(12A)^xv^	3.3551	H(16A)···H(9A)^xiv^	3.2426
C(15)···H(16A)^iii^	3.3656	H(16A)···H(9A)^vi^	3.4014
C(15)···H(16B)^iii^	3.4736	H(16A)···H(9B)^vi^	3.1460
C(16)···H(8B)^viii^	3.5151	H(16A)···H(10)^xvi^	3.0514
C(16)···H(10)^xvi^	3.0002	H(16A)···H(11A)^vi^	2.8206
C(16)···H(11A)^vi^	3.5854	H(16A)···H(11B)^xvi^	2.9551
C(16)···H(11B)^xvi^	3.4476	H(16A)···H(15A)^iv^	3.1351
C(16)···H(11B)^xv^	3.5454	H(16A)···H(15B)^iv^	2.7897
C(16)···H(15A)^iv^	3.0775	H(16B)···O(1)^viii^	3.5118
C(16)···H(15B)^iv^	3.1951	H(16B)···C(8)^viii^	3.3736
H(2)···C(6)^iii^	3.3092	H(16B)···C(10)^xvi^	3.1033
H(2)···C(15)^ii^	3.5201	H(16B)···C(11)^xvi^	3.4832
H(2)···H(6)^iii^	3.3350	H(16B)···C(12)^xvi^	3.2856
H(2)···H(15B)^ii^	2.5642	H(16B)···C(15)^iv^	3.4736
H(2)···H(16A)^ix^	3.3954	H(16B)···H(8A)^viii^	3.1585
H(5)···O(1)^viii^	2.8414	H(16B)···H(8B)^viii^	2.7043
H(5)···O(3)^iv^	3.5191	H(16B)···H(10)^xvi^	2.3527
H(5)···C(1)^viii^	3.1796	H(16B)···H(11B)^xvi^	3.1149
H(5)···C(3)^iv^	3.3346	H(16B)···H(12A)^xvi^	2.7473
H(5)···C(6)^viii^	3.1287	H(16B)···H(12A)^xv^	3.3943
H(5)···C(7)^viii^	3.0449	H(16B)···H(15A)^iv^	2.7897
H(5)···H(6)^viii^	2.9456	H(16B)···H(15B)^iv^	3.3623
H(5)···H(6)^i^	3.2433		
			
C(7)—O(2)—C(8)	116.68 (12)	H(8A)—C(8)—H(8B)	109.475
C(3)—O(3)—C(9)	116.11 (9)	H(8A)—C(8)—H(8C)	109.468
C(4)—O(4)—C(13)	117.33 (10)	H(8B)—C(8)—H(8C)	109.472
C(2)—C(1)—C(6)	120.17 (12)	O(3)—C(9)—H(9A)	109.948
C(2)—C(1)—C(7)	120.42 (11)	O(3)—C(9)—H(9B)	109.953
C(6)—C(1)—C(7)	119.40 (11)	C(10)—C(9)—H(9A)	109.961
C(1)—C(2)—C(3)	120.30 (12)	C(10)—C(9)—H(9B)	109.956
O(3)—C(3)—C(2)	124.69 (11)	H(9A)—C(9)—H(9B)	108.328
O(3)—C(3)—C(4)	115.80 (10)	C(9)—C(10)—H(10)	116.366
C(2)—C(3)—C(4)	119.50 (11)	C(11)—C(10)—H(10)	116.367
O(4)—C(4)—C(3)	115.03 (11)	C(12)—C(10)—H(10)	116.375
O(4)—C(4)—C(5)	125.26 (12)	C(10)—C(11)—H(11A)	117.777
C(3)—C(4)—C(5)	119.71 (12)	C(10)—C(11)—H(11B)	117.773
C(4)—C(5)—C(6)	120.21 (12)	C(12)—C(11)—H(11A)	117.769
C(1)—C(6)—C(5)	120.09 (12)	C(12)—C(11)—H(11B)	117.772
O(1)—C(7)—O(2)	123.48 (12)	H(11A)—C(11)—H(11B)	114.909
O(1)—C(7)—C(1)	125.10 (12)	C(10)—C(12)—H(12A)	117.744
O(2)—C(7)—C(1)	111.42 (11)	C(10)—C(12)—H(12B)	117.746
O(3)—C(9)—C(10)	108.69 (9)	C(11)—C(12)—H(12A)	117.745
C(9)—C(10)—C(11)	116.52 (11)	C(11)—C(12)—H(12B)	117.747
C(9)—C(10)—C(12)	119.44 (11)	H(12A)—C(12)—H(12B)	114.871
C(11)—C(10)—C(12)	59.78 (9)	O(4)—C(13)—H(13A)	110.344
C(10)—C(11)—C(12)	59.97 (9)	O(4)—C(13)—H(13B)	110.344
C(10)—C(12)—C(11)	60.25 (9)	C(14)—C(13)—H(13A)	110.360
O(4)—C(13)—C(14)	106.85 (12)	C(14)—C(13)—H(13B)	110.358
C(13)—C(14)—C(15)	117.46 (14)	H(13A)—C(13)—H(13B)	108.585
C(13)—C(14)—C(16)	117.91 (14)	C(13)—C(14)—H(14)	116.594
C(15)—C(14)—C(16)	59.51 (12)	C(15)—C(14)—H(14)	116.598
C(14)—C(15)—C(16)	60.61 (12)	C(16)—C(14)—H(14)	116.603
C(14)—C(16)—C(15)	59.89 (12)	C(14)—C(15)—H(15A)	117.706
C(1)—C(2)—H(2)	119.856	C(14)—C(15)—H(15B)	117.711
C(3)—C(2)—H(2)	119.846	C(16)—C(15)—H(15A)	117.702
C(4)—C(5)—H(5)	119.900	C(16)—C(15)—H(15B)	117.720
C(6)—C(5)—H(5)	119.892	H(15A)—C(15)—H(15B)	114.826
C(1)—C(6)—H(6)	119.947	C(14)—C(16)—H(16A)	117.785
C(5)—C(6)—H(6)	119.960	C(14)—C(16)—H(16B)	117.794
O(2)—C(8)—H(8A)	109.469	C(15)—C(16)—H(16A)	117.781
O(2)—C(8)—H(8B)	109.474	C(15)—C(16)—H(16B)	117.788
O(2)—C(8)—H(8C)	109.470	H(16A)—C(16)—H(16B)	114.905
			
C(8)—O(2)—C(7)—O(1)	−2.4 (2)	C(2)—C(3)—C(4)—O(4)	179.31 (11)
C(8)—O(2)—C(7)—C(1)	177.99 (12)	C(2)—C(3)—C(4)—C(5)	−1.41 (19)
C(3)—O(3)—C(9)—C(10)	160.99 (10)	O(4)—C(4)—C(5)—C(6)	179.71 (12)
C(9)—O(3)—C(3)—C(2)	11.35 (17)	C(3)—C(4)—C(5)—C(6)	0.52 (20)
C(9)—O(3)—C(3)—C(4)	−167.98 (11)	C(4)—C(5)—C(6)—C(1)	0.69 (20)
C(4)—O(4)—C(13)—C(14)	177.72 (12)	O(3)—C(9)—C(10)—C(11)	153.94 (11)
C(13)—O(4)—C(4)—C(3)	177.39 (11)	O(3)—C(9)—C(10)—C(12)	85.31 (14)
C(13)—O(4)—C(4)—C(5)	−1.83 (19)	C(9)—C(10)—C(11)—C(12)	−110.19 (13)
C(2)—C(1)—C(6)—C(5)	−1.01 (19)	C(9)—C(10)—C(12)—C(11)	105.34 (13)
C(6)—C(1)—C(2)—C(3)	0.09 (19)	C(11)—C(10)—C(12)—C(11)	0.0
C(2)—C(1)—C(7)—O(1)	175.66 (13)	C(12)—C(10)—C(11)—C(12)	0.0
C(2)—C(1)—C(7)—O(2)	−4.76 (17)	C(10)—C(11)—C(12)—C(10)	0.0
C(7)—C(1)—C(2)—C(3)	−179.24 (12)	O(4)—C(13)—C(14)—C(15)	−76.77 (16)
C(6)—C(1)—C(7)—O(1)	−3.7 (2)	O(4)—C(13)—C(14)—C(16)	−144.94 (14)
C(6)—C(1)—C(7)—O(2)	175.90 (12)	C(13)—C(14)—C(15)—C(16)	−107.83 (16)
C(7)—C(1)—C(6)—C(5)	178.33 (12)	C(13)—C(14)—C(16)—C(15)	107.08 (17)
C(1)—C(2)—C(3)—O(3)	−178.20 (12)	C(15)—C(14)—C(16)—C(15)	0.0
C(1)—C(2)—C(3)—C(4)	1.11 (19)	C(16)—C(14)—C(15)—C(16)	0.0
O(3)—C(3)—C(4)—O(4)	−1.31 (17)	C(14)—C(15)—C(16)—C(14)	0.0
O(3)—C(3)—C(4)—C(5)	177.96 (11)		

Symmetry codes: (i) *x*+1/2, −*y*+3/2, −*z*+1; (ii) −*x*+3/2, −*y*+1, *z*+1/2; (iii) *x*+1, *y*, *z*; (iv) *x*−1, *y*, *z*; (v) −*x*+5/2, −*y*+1, *z*+1/2; (vi) −*x*+3/2, −*y*+1, *z*−1/2; (vii) −*x*+5/2, −*y*+1, *z*−1/2; (viii) *x*−1/2, −*y*+3/2, −*z*+1; (ix) −*x*+1/2, −*y*+1, *z*+1/2; (x) *x*−1/2, −*y*+1/2, −*z*+1; (xi) −*x*+1, *y*−1/2, −*z*+1/2; (xii) *x*+1/2, −*y*+1/2, −*z*+1; (xiii) −*x*+2, *y*−1/2, −*z*+1/2; (xiv) −*x*+1/2, −*y*+1, *z*−1/2; (xv) −*x*+2, *y*+1/2, −*z*+1/2; (xvi) −*x*+1, *y*+1/2, −*z*+1/2.

### Hydrogen-bond geometry (Å, °)

**Table:**

*D*—H···*A*	*D*—H	H···*A*	*D*···*A*	*D*—H···*A*
C12—H12B···O3^iii^	0.99	2.55	3.4073 (18)	145

Symmetry codes: (iii) *x*+1, *y*, *z*.
